# Exploring the perspectives of selectors and collecters of trial outcome data: an international qualitative study

**DOI:** 10.1186/s12874-023-02054-9

**Published:** 2023-10-11

**Authors:** Heidi R. Green, Annabel Dawson, Adel Elfeky, David Pickles, Shaun Treweek, Katie Gillies

**Affiliations:** 1 Health Services Research Unit, University of Aberdeen, Aberdeen, UK; 2 COUCH Health, Manchester, UK; 3 Public contributor, Durham, UK; 4 Division of Health Sciences, Warwick Medical School, University of Warwick, Warwick, UK; 5 Rheumatology department, Leeds Teaching Hospitals NHS Trust, Leeds, UK

**Keywords:** Randomised controlled trials, Outcome selection, Outcome collection, Data collection, Public and patient involvement, Research waste

## Abstract

**Introduction:**

Selecting and collecting data to support appropriate primary and secondary outcomes is a critical step in designing trials that can change clinical practice. In this study, we aimed to investigate who contributes to the process of selecting and collecting trial outcomes, and how these people are involved. This work serves two main purposes: (1) it provides the trials community with evidence to demonstrate how outcomes are currently selected and collected, and (2) it allows people involved in trial design and conduct to pick apart these processes to consider how efficiencies and improvements can be made.

**Methods:**

One-with-one semi-structured interviews, supported by a topic guide to ensure coverage of key content. The Framework approach was used for thematic analysis of data, and themes were linked through constant comparison of data both within and across participant groups. Interviews took place between July 2020 and January 2021. Participants were twenty-nine international trialists from various contributor groups, working primarily on designing and/or delivering phase III pragmatic effectiveness trials. Their experience spanned various funders, trial settings, clinical specialties, intervention types, and participant populations.

**Results:**

We identified three descriptive themes encompassing the process of primary and secondary outcome selection, collection, and the publication of outcome data. Within these themes, participants raised issues around the following: 1) Outcome selection: clarity of the research question; confidence in selecting trial outcomes and how confidence decreases with increased experience; interplay between different interested parties; how patients and the public are involved in outcome selection; perceived impact of poor outcome selection including poor recruitment and/or retention; and use of core outcome sets. 2) Outcome collection: disconnect between decisions made by outcome selectors and the practical work done by outcome collectors; potential impact of outcome measures on trial participants; potential impact on trial staff workload; and use of routinely collected data. 3) Publication of outcome data: difficulties in finding time to write and revise manuscripts for publication due to time and funding constraints. Participants overwhelmingly focused on the process of outcome selection, a topic they talked about unprompted. When prompted, participants do discuss outcome collection, but poor communication between selectors and collectors at the trial design stage means that outcome selection is rarely linked with the data collection workload it generates.

**Discussion:**

People involved in the design and conduct of trials fail to connect decisions around outcome selection with data collection workload. Publication of outcome data and effective dissemination of trial results are hindered due to the project-based culture of some academic clinical trial research.

**Supplementary Information:**

The online version contains supplementary material available at 10.1186/s12874-023-02054-9.

## Introduction

Randomised controlled trials (hereafter referred to as trials) are one of the most effective ways to determine the effectiveness of interventions in healthcare. Yet they can be complex to design and conduct, and expensive to run.

Selecting appropriate primary and secondary outcomes is a critical step in designing trials that have the potential to change clinical practice [1]. There are myriad ways that outcomes can be chosen badly; Heneghan et al. named these as: (1) surrogate outcomes, (2) composite outcomes, (3) subjective outcomes, (4) complex scales, and (5) lack of relevance to patients and decision makers [2]. When outcomes are poorly selected, trial teams risk wasting huge amounts of time and money on a trial that fails to translate into clinical benefits for patients. In addition, it is unethical to collect data that will not be used, and if unused data are deemed ‘personal’ then this practice may also be unlawful.

A 2014 research priority-setting exercise involving UK Directors of Clinical Research Collaboration registered Clinical Trials Units, named ‘*Choosing appropriate outcomes to measure*’ as one of the top three priorities for trials methodological research [3]. This demonstrates just how difficult the complex process of trial outcome selection is for many. Since then, researchers have published case studies and guidance with the aim of increasing the literature to support trialists in the process of outcome selection [3, 4], but ultimately relatively little is understood about the components of outcomes that are critical to decision-making [3].

Following selection, these outcomes must be operationalised to provide measurement tools that enable trial teams to collect data to support them. In addition to the work done by participants to provide data, the trial team must create data collection tools, build data management systems, follow-up with sites and conduct data cleaning processes in the inevitable cases of missing or spurious data, and that’s before they tackle data analysis. It is therefore not surprising that data collection is estimated to consume well over 30% of all working hours spent on trials [5].

A study of 116 US-based Phase III trials published in 2015, found that extraneous procedures in clinical trials are costing the pharmaceutical industry up to $5 billion each year [6]. These ‘extraneous procedures’ are almost entirely linked to trial outcomes, with the study authors finding that tests not linked to key efficacy or safety trial endpoints can account for as much as 20% of a trial’s budget. The report suggested that if trial sponsors directly tie the design of their protocols to their primary endpoints, sponsors of Phase III trials could save an average of $1.7 million per trial on the direct costs of lab tests or participant questionnaires [6].

Recently 96 Cochrane systematic reviews that included 1659 trials conducted in 84 countries were analysed. Of the 1640 trials that provided risk of bias information, 62% were deemed to be at high risk of bias and 30% has risk of bias information that was judged to be unclear [7]. The proportion of drug trials that were high risk of bias was 54%, lower than the 66% seen for non-drug trials, suggesting perhaps that industry-led trials tend to be more methodologically sound than academic trials. The study did not assess which domains drove the overall risk of bias judgements, but as three of the six domains in Cochrane RoB 1.0 relate to outcomes (blinding of participants, personnel and outcome assessors; incomplete outcome data; selective outcome reporting), and three of the five domains in Cochrane RoB 2.0 (incomplete outcome data; outcome measurement; selective reporting), it is fair to assume that poor outcome selection, collection, and reporting were the reason for at least some of the high and uncertain judgements [7]. The study authors’ low estimate of the cost of ‘bad’ trials (i.e. those at high risk of bias) was £726 million, their high estimate was over £8 billion [7].

Perhaps these increases in time, cost, and effort, may be justified, or at least not wasted, if the additional data were being used. Unfortunately, this is not the case.

A study of 2711 trials included in the main comparison of 290 Cochrane systematic reviews, found that most of the trials did not contribute to all meta-analyses of the most important outcomes, mainly due to inadequate planning or incomplete reporting of outcomes [8]. Such waste could have been partially avoided for 63% of the trials, and totally avoided for 30%^8^.

A Cochrane systematic review comparing entries in trial registries to the reports published at the end of the trial found that between 10 and 18% of primary outcome data, and 44% of secondary outcome data were not published [9].

In a review of all trials submitted to a German ethics committee between 2000 and 2002, Kirkham et al. found that of the two and half million items of outcome data collected from participants across 308 trials, only 47% were published in full [10]. In addition, a review of cancer clinical trials completed by the Ontario Clinical Oncology Group between 2003 and 2012 revealed that between 186 and 1035 data items were collected per participant, but a median of 82% of collected data were not reported in associated publications [5]. The substantial volume of unreported data from case report forms had commonalities, with several categories of data rarely, or never, used in publications [5]. Of the 18 data categories, a total of 4% or less of collected items were eventually reported in 8 categories, and the biggest data category was ‘ID/privacy characteristics’, with a median of 91 data items collected per participant per trial, 0% of which were reported in publications [5]. The investment in non-outcome data is echoed by Crowley et al., who categorised data from 18 trials run in the UK and Ireland, and found that participant identifiers and demographic data represented 32.4% (median), or 36.5% (mean) of all data items collected [11].

The ultimate aim of clinical research is to improve the health of patients [2]. Poorly selected, impractically collected, unanalysed, misinterpreted, and unreported outcome data is unethical, and represents a substantial volume of global research waste. Unfortunately, the waste in terms of time and money is incomparable to the missed opportunities to improve the health of patients and the care that they receive.

The Trial Forge initiative (https://www.trialforge.org) is an evidence-based approach to designing, running, analysing, and reporting trials. Trial Forge aims to look across all trial processes with the intention of trying to improve them all, even if it’s just by a tiny amount, because these gains will add up when combined. The work presented in this manuscript is part of the Trial Forge initiative, and aims to improve trial design with a particular focus on primary and secondary outcomes.

In this semi-structured one-with-one interview study, we aimed to investigate who contributes to the process of selecting and collecting trial outcomes, and how those people are involved. This work serves two main purposes: (1) it provides the trials community with evidence to demonstrate who contributes to the process of outcome selection and collection, and (2) it allows people involved in the design and conduct of trials to pick apart these processes to consider how efficiencies can be made.

## Methods

This qualitative study is part of a larger project called ORINOCO (Optimising Resource-use IN Outcome Collection). ORINOCO had three phases; the first two phases aimed to increase awareness amongst trialists of how data collection effort is distributed across outcomes, and this work has been described elsewhere [12]. This paper addresses the third phase of ORINOCO.

### Sampling and study procedure

We sought to identify potential interview participants from a range of sources. Our interview study was advertised via email newsletters through a range of existing networks: Trial Forge collaborators, Medical Research Council Trial Methodology Hubs, UK Clinical Research Collaboration Registrered Trials Units, and relevant mailing lists (Trial Forge, Ireland’s Health Research Board-Trials Methodology Research Network, UK Trial Managers’ Network). The research team also shared information about the study within their own professional networks including on social media sites LinkedIn and Twitter. Interested individuals were encouraged to contact the Research Fellow (HG) for more information, and were then sent a copy of the participant information leaflet (Additional File 1). Potential participants were given an opportunity to ask questions about the study before an interview was scheduled, and participants were asked to sign and email or post the completed consent form before the interview took place.

We focussed on including study participants who worked primarily on designing and/or delivering Phase III pragmatic effectiveness trials, i.e., trials designed to provide a definitive answer to their research question, though some of our participants did have additional experience of early-phase exploratory trials and were able to contrast these with their experience of Phase III trials. We aimed to include a range of participants based on their experience and trial portfolios; including experience with a variety of funders (e.g., public, private, and third sector), trial settings (primary, secondary, or tertiary care settings), clinical specialties, intervention types (e.g., investigational medicinal product, licensed drug, surgical technique, medical device, and behavioural and lifestyle change interventions), and participant populations. We monitored the experience of participants as they were recruited and were therefore able to tailor messaging and recruitment advertising to ensure gaps in experience were filled throughout the recruitment period. This resulted in everyone who showed an interest in participation being interviewed.

### Data collection

One-with-one interviews were conversational in style and semi-structured in nature, supported by a topic guide to ensure coverage of key content. All interviews were conducted by HG, a Research Fellow with expertise in trial recruitment and methods research and white British woman, aged 28 at the time of the interviews. Participants were made aware that HG does not have a clinical background and has never been tasked with selecting or collecting trial outcome data.

At the start of each interview, participants were encouraged to discuss their current role and its day-to-day responsibilities. Following this, the topic guide was used to support discussion centred around primary and secondary outcome selection and collection. The topic guide (Additional file 2) was refined throughout the study, and HG took notes after each interview to assist analysis and interpretation.

Data collection took place between July 2020 and January 2021, and interviews were conducted over the telephone, or using online video chat platforms (e.g., Zoom, Microsoft Teams) based on interviewee preference.

Following each interview, the audio file was securely sent to an external transcription service approved by the University of Aberdeen. Interviews were transcribed verbatim in advance of analysis.

### Data analysis

Data were analysed using the Framework method, a type of thematic analysis developed with an initial focus on applied policy research [13–15]. The Framework method is particularly useful for pragmatic research with clear objectives, a pre-defined sample, tight timeframes, and teams with varying levels of qualitative research experience. For these reasons, the Framework method has been widely and successfully used for applied health services research [16–21].

HG coded three transcripts using an open coding approach to develop a working analytical framework. KG (a white British woman in her early 40s with expertise in participant centred clinical trials research) then independently reviewed three different transcripts along with the working analytical framework. Coding and themes were discussed by HG and KG to agree the analytical framework that would be applied to all transcripts, as well as the details of how this framework would be operationalised. No differences or disagreements remained after discussion, if this had been the case we would have brought in another member of the project team to resolve them. HG applied the framework to all transcripts using NVivo software to help organise the data into codes that could then be compared within and across participant characteristics (participant group and location). Following analysis, HG selected participant quotes to illustrate specific themes within our study findings. Quotes presented here have been anonymised to protect confidentiality.

## Results

### Participants

We invited 64 individuals to secure 29 interviews with international trialists from a range of contributor groups (Table 1). Interviews lasted between 22 min and 1 h 35 min (median: 55 min).

We purposively recruited participants to gather views from a range of contributor groups, geographic locations, and genders. We interviewed participants from seven contributor groups; Chief Investigator (all participants in this group were also Clinicians), Ethics Committee Member, Funder, patient and public involvement (PPI) Partner, Statistician, Trial Conduct Expert (these participants currently have strategic oversight roles in trial delivery, building on roles held previously such as Trial Manager and Research Nurse), Trial Manager. The only group we intended to recruit and did not, were representatives from Sponsors. We contacted three people in the UK from this group (all from academic institutions) and all declined to take part as they did not feel they could contribute, explaining that they relied on Chief Investigators and their teams to make appropriate decisions on outcome selection and collection.

**Table 1:** Characteristics of interviewees

Interviewee characteristics (N = 29)	
**Contributor group**	
Chief Investigator (all participants in this group were also Clinicians)	8
Ethics Committee Member	3
Funder	3
Patient and Public Involvement Partner	4
Statistician	3
Trial Conduct Expert	5
Trial Manager	3
**Location**	
Australia	2
Belgium	1
Canada	1
Denmark	1
Germany	2
Ireland	2
Netherlands	1
Switzerland	1
UK	16
USA	2
**Gender**	
Man	16
Woman	13
Non-binary	0

### Overview of findings

Three broad descriptive themes were identified in the data; we begin with findings encompassing the process of primary and secondary *outcome selection*, then follow on with findings relevant for primary and secondary *outcome collection*, finishing up with a short theme discussing *publication of outcome data* which links to academic research culture.

Interviewees overwhelmingly focussed on outcome selection. They discussed this process automatically when the general topic of primary and secondary outcomes was introduced, and required additional prompts to discuss data collection. This lean towards selection over collection is reflected in the volume of themes presented.

## Theme 1: primary and secondary outcome selection

Within the primary and secondary outcome selection theme, we identified seven sub-themes: (1) clarity of the research; (2) confidence in selecting trial outcomes; (3) interplay between different groups; (4) patient and public involvement; (5) perceived impact of poor outcome selection; (6) use of experiential evidence; and (7) use of core outcome sets.

### Clarity of the research

Participants from every contributor group discussed the clarity of the research question when asked about the process of selecting trial outcomes. Interviewees explained that without a clear research question, additional outcomes can be introduced.

*“I think one of the key issues is about being clear about what your trial is seeking to achieve. And therefore, being clear about what the primary outcome is and sometimes you find in trials that when there’s a lack of clarity about exactly what question the trial is going to answer, you know, when that gets a bit fuzzy then you find the outcomes tend to proliferate.”* (Chief Investigator and Clinician, UK, Participant 17).

### Confidence in selecting trial outcomes

The importance of outcome selection was discussed by all participants, with that importance seeming to weigh on participants, particularly Chief Investigators who are ultimately responsible for a trial. This weight is inversely related to confidence in selecting trial outcomes: the more experience and work that people build up in this area, the “*less and less*” confident they feel in their decisions (Chief Investigator and Clinician, Denmark, Participant 20).

*“I’m confident that I know the process is really complex and not simple, and that my views and of what’s important may differ to other people’s perceptions. So, I guess how confident and I? I’m confident that I know that this isn’t simple, but I don’t have all the answers so that’s probably as confident as I am.”* (Chief Investigator and Clinician, Ireland, Participant 4).

### Interplay between different groups

The interplay between contributor groups was a significant theme within our interviews. Participants described the process of gathering trial contributors together to thrash out the design of a trial. These discussions happen before the trial is funded as they feed into building a competitive grant application. As one Trial Manager (UK, Participant 11) put it, *“an outcome is met via the consensus of a room”*.

The ultimate responsibility for these trials falls on the Chief Investigator, historically a Clinician. Participants from other contributor groups described the process of Clinicians often adding outcomes into these discussions. Participant groups tasked with dealing with an increasing volume of data explained their need to compromise to ensure that Clinicians are happy, while also ensuring that the team is able to handle the volume of data being generated.

*“The worst thing in the world is an investigator coming along and saying, “Wouldn’t it just be great if we could just add on a measurement of blah?” and as a CTU director, then your heart used to always sink at that point and go, “Oh god, here we go”, it’s easy for an investigator to say that, and the sort of incremental effort on the part of the investigator himself, or herself, is minimal, and what’s not seen is the hidden work in all of that.”* (Chief Investigator and Clinician, UK, Participant 17).

### Patient and public involvement

Participants discussed the importance of patient and public involvement when selecting outcomes. Some Chief Investigators talked about PPI Partners having influence in other areas of the trial, but not outcome selection. Other Chief Investigators discussed PPI Partners pushing forward what is important to them, and how the trial team then needs to translate that into a measurable outcome. The experiences of Chief Investigators contrasted, sometimes starkly, with those of the PPI Partners we interviewed.

*“I mean I think PPI members are quite good at spotting the difference between substantive and surrogate outcomes. They wouldn’t ever use those phrases, those terms. They don’t know what that means, but they know a meaningful outcome to them when they hear it and one which they go, “Well, what does that mean?””* (Chief Investigator and Clinician, UK, Participant 22).

*“The PPI group were asked, “Okay, so this is going to be the primary outcome, how do you want us to measure it? Do you want us to measure it with time on the treadmill or do you want us to measure it with an MRI?” so it was Hobson’s choice, it wasn’t really a choice, it wasn’t them saying, “What primary outcome is meaningful to you?””* (PPI Partner, UK, Participant 12).

### Perceived impact of poor outcome selection

The perceived impact of poor outcome selection was a topic that was discussed by all contributor groups. Chief Investigators described how asking participants to do *“too much”* is likely to negatively impact recruitment and retention. Both PPI Partners and Trial Conduct Experts agreed the impact of poor outcome selection was poor recruitment. However, PPI Partners connect poor recruitment to the importance of outcomes that are important to patients, and Trial Conduct Experts made the link to workload pressures.

*“You can’t run the clinical trial if you don’t get participants, and participants are going to vote with their feet, and it’s not there yet, but I think as people learn more about research, as people become more sophisticated as whatever, then people are going to say, “Well actually you know what… No, I’m not taking part in that because your primary outcome’s pants, absolutely no relevance to me so I’m not taking part in that trial”.”* (PPI Partner, UK, Participant 12).

*“The more time you spend capturing data for primary and secondary outcomes, the less patients you’re likely to recruit. We either recruit to target in the time span of a clinical trial, or we capture more data points.”* (Trial Conduct Expert, Australia, Participant 19).

### Use of experiential evidence

When asked how outcomes are selected in trials, participants discussed the importance of experience; particularly focussing on selecting outcomes and the ability to forecast potential problems with data collection. This was not only described by members of the trial team, Ethics Committee Members also highlighted their reliance on experience when reviewing studies to assess whether selected outcomes can be practically collected.

*“I mean it starts with the idea for the trial and then it has, of course, to do a lot with experience so you should always ask someone who has already done a clinical trial, or more than one clinical trial, and then you take a blank paper, and you write the outcomes on it and then you play.”* (Chief Investigator and Clinician, Germany, Participant 24).

*“We look at the practicality of collecting outcomes… They’re* [trials] *particularly prey to what I call the laundry list type of research. They are often relatively inexperienced researchers who believe that they can do a great deal more than they could do, so what they’re suggesting is impractical and quite often we have to manage that. ”* Ethics Committee Member, UK, Participant 2).

### Use of core outcome sets

Most participants talked positively about core outcome sets and the COMET initiative (https://www.comet-initiative.org), broadly viewing them as a useful way to increase efficiency and ensure trial data can be aggregated, though there was some frustration reported too. Areas of frustration spanned core outcome sets that are very long and may contain outcomes that are irrelevant in their view, and the perceived potential for core outcome sets to stifle innovation in outcome selection.

*“We go to the COMET initiative and look there to see if there is a core outcome set for this indication, and if there is a core outcome set, you’d have to have a really good reason not to include it in what you’re looking for, so that will go in by default.”* (Funder, UK, Participant 1).

*“The one thing I have a difficulty with is their core outcome sets. I think the principle of core outcome sets are really important, but the slavish adherence to core outcome sets means that for some trials the list of secondary outcomes is enormously long, because that’s the core outcome set.”* (Chief Investigator and Clinician, UK, Participant 22).

## Theme 2: primary and secondary outcome collection

Within the primary and secondary outcome collection theme, we identified four sub-themes: (1) disconnect between outcome selectors and outcome collectors; (2) potential impact on trial participants; (3) potential impact on trial teams; and (4) use of routinely collected data.

### Disconnect between outcome selectors and outcome collectors

Interview participants inexperienced with collecting data discussed the use of ‘innovative’ data collection methods. Many of the examples given explored the use of genomics and biomarkers to measure clinical outcomes. Interviewees with experience of data collection tended to be sceptical of these ‘innovative’ ways to collect data due to the lack of practicality, and perception that the process they already had in place worked.

*“Really interesting discussion, actually by a clinician, who said he’d been approached by somebody who developed a blood test designed to detect appendicitis. He said, “The problem is, that the test required its own equipment to run it.” It’s a biomarker or something like that. He said, “If I need to know if someone’s got appendicitis, just poke them in the right place in the stomach, and 99% of the time, that will tell me whether they’ve got appendicitis.””* (Ethics Committee Member, UK, Participant 2).

One Trial Conduct Expert explained that when working as a Research Nurse, she’d never had a *“direct link”* to a Statistician that she’d be working on a trial alongside. This highlights both the focus on outcome selection over collection, but also the disconnect between the people working to select outcomes, and those that collect data to support them.

*“So as a clinical trials nurse, you know, on the ground and actually capturing the data, no, we don’t often speak to statisticians. I can say that both in terms of investigator-led and industry trials, I’ve never had a direct link to a statistician.”* (Trial Conduct Expert, Australia, Participant 19).

The same participant also discussed issues that arose due to a disconnect between outcome selectors and outcome collectors. In this case, the applicability of specific outcomes to the participant population had not been considered when selecting outcome measures, leading to consistent missing data.

### Potential impact on trial participants

The potential impact of data collection on trial participants was a subject discussed by all participant groups. Trialists discussed the impact on patients and participants, whereas, PPI Partners focussed on the motivation of participants to contribute to the trial.

*“I always focus on the additional burden because most clinical trials actually expect patients to make themselves available outside even of their routine clinic visits, okay? So the complexity comes in where it’s not a young person who can just stroll in and get it done, you know? Do they have a carer? Do they have to come in? What additional resources and support are needed even before they get to the site? If it’s an elderly population, how much stuff are you asking them to complete? How tasking is it? How exhausting is it, you know?”* (Trial Conduct Expert, UK, Participant 23).

*“I hate this use of “burden” and “suffering”, because unless you’re directly… don’t put burden and suffering on to people…I think that some trial populations are very, very motivated and will be motivated to do quite a complex trial and to do quite a lot.”* (PPI Partner, UK, Participant 12).

### Potential impact on trial teams

In contrast to the impact of data collection on trial participants, the potential for data collection to have an impact on the trial team was not discussed much by our interviewees. Largely, this theme was talked about by the people that had been impacted by data collection methods; Trialists, struggling to collect data from trial participants, and Statisticians dealing with an ‘unwieldy’ dataset.

*“It was unwieldy, and the data team didn’t really want to go near it, they didn’t want to do much validation on it because it was unwieldy big, and the data set that came from it was huge. Most of it was one questionnaire and it was a very in-depth questionnaire that was like, “Have you done this? How many times have you done this? How many times have you done this with person A, B, C?”*” (Statistician, UK, Participant 10).

### Use of routinely collected data

All contributor groups discussed routinely collected data when asked about outcome data collection. There were various views presented with a consistent understanding that routinely collected data could contribute to a pragmatic trial, but multiple downsides to its use were also described. Chief Investigators and Trialists focussed on infrastructure and bureaucratic hurdles; and Statisticians centred on their sceptism on data quality. One PPI Partner explained that routinely collected data lack richness as information about how patients manage their own health is missing.

*“We do try to make the trials as pragmatic as possible, so that we’re reliant on routine data as much as possible, however in reality that depends on going through the infrastructure to which those outcomes are collected …We end up just coming along and saying, “Actually we want to collect this in one single place because it’s more efficient so let’s ask the patients to complete this”.”* (Chief Investigator and Clinician, Ireland, Participant 4).

*“We don’t collect data from a patient’s point of view, I have no control over what data the health service thinks is important to collect, I have no input, not me, [Name], inputting into that data, and I don’t have access to my medical notes.”* (PPI Partner, UK, Participant 12).

## Theme 3: publication of outcome data

Within the publication of outcome data theme, we identified two sub-themes: (1) factors that contribute to publication of outcome data; and (2) strategic selection of outcomes.

### Factors that contribute to publication of outcome data

Participants from all contributor groups discussed the publication of outcome data, whether in scientific journals or held in repositories such as the NIHR Journals Library. Participants cited literature that provides evidence that outcome data is often not reported in full. Chief Investigators highlighted that this is not the case with their trials. Funders explained that they are doing their best to encourage full publication of trial data, sometimes imposing sanctions to push people to do so rather than providing rewards after publication.

*“I know that when people have looked at this in the past, I know it’s something silly isn’t it? Lots of data just does not get to see the light of day. I think that that’s not the case in our trials because if we’re collecting an outcome, we report the outcome.”* (Chief Investigator and Clinician, UK, Participant 17).

*“We’ve included a sanction where reporting the trial data is compulsory because we don’t want that resource we’ve invested to be wasted. We will hold a final payment if they don’t publish a certain level of data.”* (Funder, Ireland, Participant 21).

Participants described how they intend to publish their outcome data, in some cases going so far as to allocate work by dividing related outcomes into groups and putting names to specific planned manuscripts. The same participants went on to explain that this process does not always result in outcome data being published due to the project-based nature of academic clinical trial research. This can mean that funding runs out before publications are finalised and time pressures mean that writing is delayed, or the publication abandoned entirely.

*“There’s maybe one paper, main outcome paper, published, and the rest is waiting to be analysed, the rest of the data. And then people tend to move on to other projects and do other stuff, and then it really depends on the researchers and how consistent or how persistent they are, if they’re gonna finish that or not… I think everybody overestimates how much time they have for writing.”* (Trial Conduct Expert, Netherlands, Participant 7).

### Strategic selection of outcomes

Interestingly, most participants discussed the idea of strategically selecting outcomes in an effort to increase the number of publications that a team can get in a distant way, talking about the perceived practice of other trial teams rather than their own. One participant described this strategic selection of outcomes with publications in mind as being *“fundamental to that whole house of cards that is academia”* (Chief Investigator and Clinician, UK, Participant 29).

When participants did acknowledge this activity within their own teams, the reasons behind it fell into two areas. The vast majority of trial teams explained that they wanted to maximise the efficiency of the trial by using outcome data more than once. One person described this practice as being fuelled by what the participant described as an *“intrinsic motivating factor to help out others”* (Chief Investigator and Clinician, Switzerland, Participant 25).

*“There definitely is some form of, “How many papers can we get out of this? Is there anything more we can do with this data?” but that always comes later. It’s like, “Here we’ve got the trial, the trial’s plodding along, okay, what other papers could we get from the trial?” rather than it being at the forefront when you’re planning the trial.”* (Statistician, UK, Participant 10).

*“I had a discussion with one of my co-applicants and he was shouting at me about how many outcomes I was putting in. He said it’s unrealistic and I said, “Look, it’s not just about me. It’s about building up careers.” The truth is, yes, it costs. Yes, it’s inefficient. Yes, it can blow up your whole trial if you have too many. At the same time, each of these additional outcomes is a PhD student who can write their dissertation on that.”* (Chief Investigator and Clinician, Switzerland, Participant 25).

## Discussion

### Summary of findings

Participants involved in the design and conduct of trials overwhelmingly focused on the process of outcome selection in our interviews, a topic they talked about unprompted. When prompted, participants did discuss outcome collection, but poor communication between selectors and collectors at the trial design stage means that outcome selection was rarely linked with the data collection workload it generates. The publication and effective dissemination of outcome data produced by trial teams is then limited due to the project-based culture of some academic clinical trial research.

Our findings provide insight into who is involved in outcome selection and collection activities and when, and what experiences and outside influences impact the decisions that involved parties make.

Most studies of outcomes have focused on outcome selection [22] and adherence to reporting guidance [23–25]. Our study is different. We were interested in the practical process of selecting and, especially, collecting outcome data. The latter in particular is a new perspective and, as we found, few trialists we interviewed really think about the link between outcome selection and the work of data collection that their choices lead to. If this additional work led to the generation of useful data, perhaps the disconnect could be overlooked, but we know that this is not the case. Both primary and secondary outcome data are poorly reported [9, 10], stifling the potential impact of trials on clinical practice.

### Selecting outcomes with impact

All of our participants linked outcomes to impact. Impact was conceptualised somewhat differently across contributors, but was described variously as a trial that has meaning for patients, a trial that recruits and retains sufficient participants, the ability to see a difference between intervention(s) and comparator(s), and the capacity for results to be adopted into clinical practice. The process of attaining impact was threaded through the many of themes raised during discussion of outcome selection and can be most easily summarised as trial teams want to select outcomes that provide evidence that will ultimately make a difference to patients.

Not all trial team members feel confident that they can do this. This thread ties in with the reliance on experience and counterintuitively perhaps, we found that the more experience Trialists and Chief Investigators have in doing trials and exploring potential outcomes, the less confident they feel about outcome selection. As individuals become more aware of their research area and the potential for more effective outcomes to be developed, more doubt sets in. This contrasts with the perspectives and perception of people who do not have to select trial outcomes, such as ethics committee members and funders. These groups have greater confidence in more experienced trial teams, viewing them as a safe pair of hands.

### Disconnect between outcome selectors and outcome collectors

Operationalising impact through the lens of outcome selection was often done through communication and all study participants discussed this, usually focused on what happens during the process of pulling together an application for grant funding. Overwhelmingly, participants referred to the group involved in this process as ‘the whole trial team’, but when prompted to explain who was involved in those conversations there was a clear disconnect between the outcome selectors and the outcome collectors. During an interview, one participant realised that they have never seen a Research Nurse (an outcome collector) take part in one of these conversations; the participant looked visibly confused before pausing and recognising that a Research Nurse would ‘*bring a lot to the table if they were in that room*’. In an interview with a Research Nurse who regularly collected trial outcome data, it was clear that the nurse had never had a ‘*direct link’* with a Statistician. By not involving outcome collectors in discussions about outcome selection, the link between outcome and data collection workload is unlikely to be well articulated. This highlights the need for trial teams to be aware not only that communication is happening, but that an awareness of *who* is involved in that communication is important too.

### Patient and public involvement

Patient and public involvement was a theme commonly discussed during our interviews, and participants were careful to highlight the involvement of PPI contributors in conversations about outcome selection. Intriguingly, the only divergence in views that we saw by country was in this area. Participants with UK-based experienced shared nuanced views spanning positives about importance of co-production and meaningful involvement, alongside more negative perceptions and experiences about power, control, and unspoken hierarchies between PPI contributors and the academics working with them. Views from participants with experience outside the UK were less nuanced, with many reporting that the process of involving patients and the public in trias in their locations was less established than in the UK.

The power dynamics discussed by our UK-based participants aligned with the views being shared by different participant groups. Chief Investigators in the UK were keen to discuss the importance of including patients in conversations about the trial, with some explicitly discussing outcome selection in this process, whereas an experienced PPI partner shared their frustration with being presented with ‘*Hobson’s choice’*. Hobson’s choice refers to a ‘free choice’ in which only one thing is actually offered, an illusion that multiple choices are available. In this scenario the PPI contributor is describing being asked between outcome A or B, rather than being asked *“what primary outcome is meaningful to you?”*. This frustration led to further discussion about the emotional toll that involvement has on PPI contributors, particularly around feelings of lack of control, negotiating aspects of identity in terms of being a patient and a research partner, and reliving past trauma linked to experiences with the healthcare system. These experiences have been reported in UK-based health research in recent years, particularly in mental health research [23–28], but the emotional investment and impact of involvement with trial methodology research is less well documented.

Not only do our findings add to the literature reporting unequal power dynamics from patient perspectives, our interviewees also shared how established funding models and the academic systems that trials are being conducted within has an impact on the publication of trial outcomes.

### Publication of outcome data

The pressure of running trials within an academic environment is apparent to anyone working in this area. This extends beyond the lifetime of the trial with the added pressure on researchers to write and publish manuscripts after the trial has ended and some staff have moved on to other trials or jobs. In most cases the funding period will also have ended due to a project-based working environment in academic clinical trial research with employment contracts linked to the completion of specific projects [29, 30]. Once in new posts, new responsibilities and time pressures steadily move the writing of previous manuscripts down to-do lists [30], a factor that contributes to the pile of unpublished research discussed in the Introduction [9, 10]. Our participants, particularly those working on trials based in the UK, described how their teams would make sure to publish one main paper detailing the trial’s main outcome data, but other planned papers, including those involving secondary outcome data, would remain unpublished. Whether or not these additional papers materalise was perceived to be down to how persistent researchers are, with one participant explaining that ‘*everybody overestimates how much time they have for writing’*.

A Funder representative based in Ireland explained that they have introduced a sanction to push people to disseminate trial results rather than providing rewards after publication. We did not interview trialists that referenced this particular funder’s processes, but a UK-based Chief Investigator and Clinician did reference the trial report that is expected at the end of NIHR-funded trials. NIHR guidance notes that key outcomes and trial protocols should be made publicly available within 12-months of primary study completion [31]. Our interviewees frequently made reference to publishing outcomes in trial reports, suggesting that the expectation of a report from trial funders does have an impact on the dissemination of outcome data following trial completion.

### Strengths and limitations

A significant strength of this study is the diversity within our sample; we included Chief Investigators, Ethics Committee Members, PPI Partners, Statisticians, Trialists, and Trial Managers with experience in trials based in ten countries around the world. Our participants reported differences in patient and public involvement between the UK and other countries, largely due to infrastructure availability, but other themes showed common experiences within the trials community. Our findings reflect the experiences of trialists working to design and deliver trials in various environments, both with and without a trials unit, and across clinical areas. The experiences and perceptions of the process of outcome selection and collection were consistent, suggesting that our findings will apply to trialists outside of our immediate participant population.

There are three limitations to our study; (1) the way we approached and invited people to take part, (2) the lack of representation from Data Managers, and (3) that most participants were involved and experienced with investigator-led research.

We approached participants via a range of trial methodology-focussed networks and mailing lists. The research team also shared information about the study within their own professional networks including on social media sites LinkedIn and Twitter. Both the study team and the networks we are linked with are likely aware of the drive for efficient trials and engaged to some degree with trials methods research. We approached 64 individuals to conduct 29 interviews and the main reason given for declining to take part was lack of time, so potential participants were fairly engaged, and likely interested in outcome selection and collection. That said, our findings demonstrate that even those engaged with trial methods research are relying on experiential evidence, and potentially doubtful of their ability to select and collect outcomes that will ultimately contribute to a trial with impact.

The second limitation of our study ties into our method of approach; we did not manage to recruit and interview any Data Managers. We know that Data Managers are at the forefront of processing trial data, and their perspective would have provided valuable insights.

The third, minor limitation of our study was that most participants were experienced with investigator-led research. This model is how much of the funding available for pragmatic trials is awarded particularly in the UK, and this is reflected in our sample. It is not clear how or if our findings would translate to trial teams working in less pragmatic, more exploratory trials, or trials that rely on funding methods that do not use investigator-led models. In addition, we purposely included study participants who worked primarily on designing and/or delivering Phase III pragmatic effectiveness trials. This could be viewed as a strength and a limitation depending on what the reader is hoping to find in this manuscript; our focus was to explore a specific type of trial in order for improvements in this type of trial to be made in the future. Some of our participants did have additional experience of early-phase exploratory trials and were able to contrast these with their experience of Phase III trials, but additional interviews and a re-focussed topic guide would be needed for us to confidently present findings on how the process of outcome selection and collection differs between exploratory and pragmatic trials.

### Implications for practice

Outcome selection and collection is at the core of the work required to design and deliver a successful trial but currently trial teams disproportionately focus on outcome selection. Our study highlights a disconnect between outcome selectors and collectors, and provides evidence that this disconnect, at least in part, is to blame for unrealistic outcome collection expectations. This needs to change. At the beginning of the trial design process, all parties that will be involved in the design and delivery of the trial should be represented. Often the specific people that would ultimately be tasked with trial delivery will not yet be working in post (e.g. Trial Managers, Research Nurses), if this is the case then someone else with the required expertise and practical knowledge should be involved. Including all relevant groups in discussions about outcome selection early in the process of trial design will ensure that these decisions are made with the practicalities of outcome data collection in mind.

Our findings highlight that including people in discussions about trial design is not enough. We found that PPI Partners are routinely invited into these spaces, but not given a meaningful choice about what outcomes should be selected to produce trial results that have the potential to impact their lives. We encourage the involvement of PPI Partners at all stages of the trial, but involvement is not enough. PPI Partners should be asked open questions and afforded space to share their experiences and hopes for the trial, not asked which of option A or B they would prefer.

Training is a topic that is often highlighted as a solution when PPI is not having the intended impact, and we agree that training would help. In this case, we propose that it is researchers and trial teams that would benefit from training rather than PPI Partners; the Edinburgh Clinical Research Facility [32], Imperial College London’s Faculty of Medicine [33], and the NIHR School for Primary Care Research [34] all offer resources and training for researchers. The need for researchers to be trained on how to effectively facilitate meaningful patient and public involvement is backed up by recent work that found that patients and healthcare professionals agreed with the choice of primary outcome made by trial teams doing late-stage trials in breast cancer management and nephrology just 28% of the time [35]. In addition, ‘PPI practices in selecting trial outcomes of importance to patients’ and ‘PPI practices in selecting how to measure trial outcomes’ were ranked second and seventh in a list of ten methodological priorities for clinical trials [36].

Hierarchies in trial teams remain, and our findings show that this is not only a moral issue, it is a problem that contributes to trials that are less likely to translate to meaningful benefits for patients.

### What should trial teams take away from this study?

We have four recommendations for trial teams to consider in order to improve and align the three outcome areas discussed in this manuscript: selection, collection, and publication. Each of these recommendations should be implemented at the design stage to ensure maximum benefit.

1. Involve **all** interested parties freely in discussions about the selection of primary and secondary outcomes.
2. While discussing trial outcome selection, involve **all** interested parties in discussions about how data will be collected.
3. Implement a full justification process for outcomes that connects not only individual outcome selection and collection, but also how the data will be managed, cleaned, analysed, and reported.
4. Be brutally honest regarding how much time you have to write. If it is not feasible to report all primary and secondary outcomes, consider whether these outcomes should be selected and collected at all. If they really are important but time is limited, be strategic with the dissemination methods that you choose; shorter, more accessible formats may be preferred routes to ensure all outcome data is reported.

## Conclusions

People involved with the process of designing trials focus their thoughts on outcome selection, but not the practicalities of outcome collection. They fail to associate decisions around outcome selection with data collection workload, due to a disconnect between outcome selectors and collectors; discussions about the trial at the design stage are overwhelmingly outcome selectors, with little representation from outcome collectors. PPI partners tend to be involved in discussions at the design stage and throughout the lifetime of the trial, but are often not given a meaningful choice when it comes to outcome selection.

Publication of outcome data and effective reporting and dissemination of trial results are hindered due to the project-based culture of some academic clinical trial research.

### Electronic supplementary material

Below is the link to the electronic supplementary material.

Supplementary Material 1

Supplementary Material 2

## Data Availability

All data analysed during this study are included in this published article [and its supplementary information files].
