# Supplementary figures and images for: Exploring the perspectives of selectors and collecters of trial outcome data: an international qualitative study
Source: BMC Med Res Methodol. 2023 Oct 11;23:229. doi: 10.1186/s12874-023-02054-9 (PMC10568821; doi:10.1186/s12874-023-02054-9)

# ORINOCO Participant information leaflet

Version 1.0, 26th November 2019.
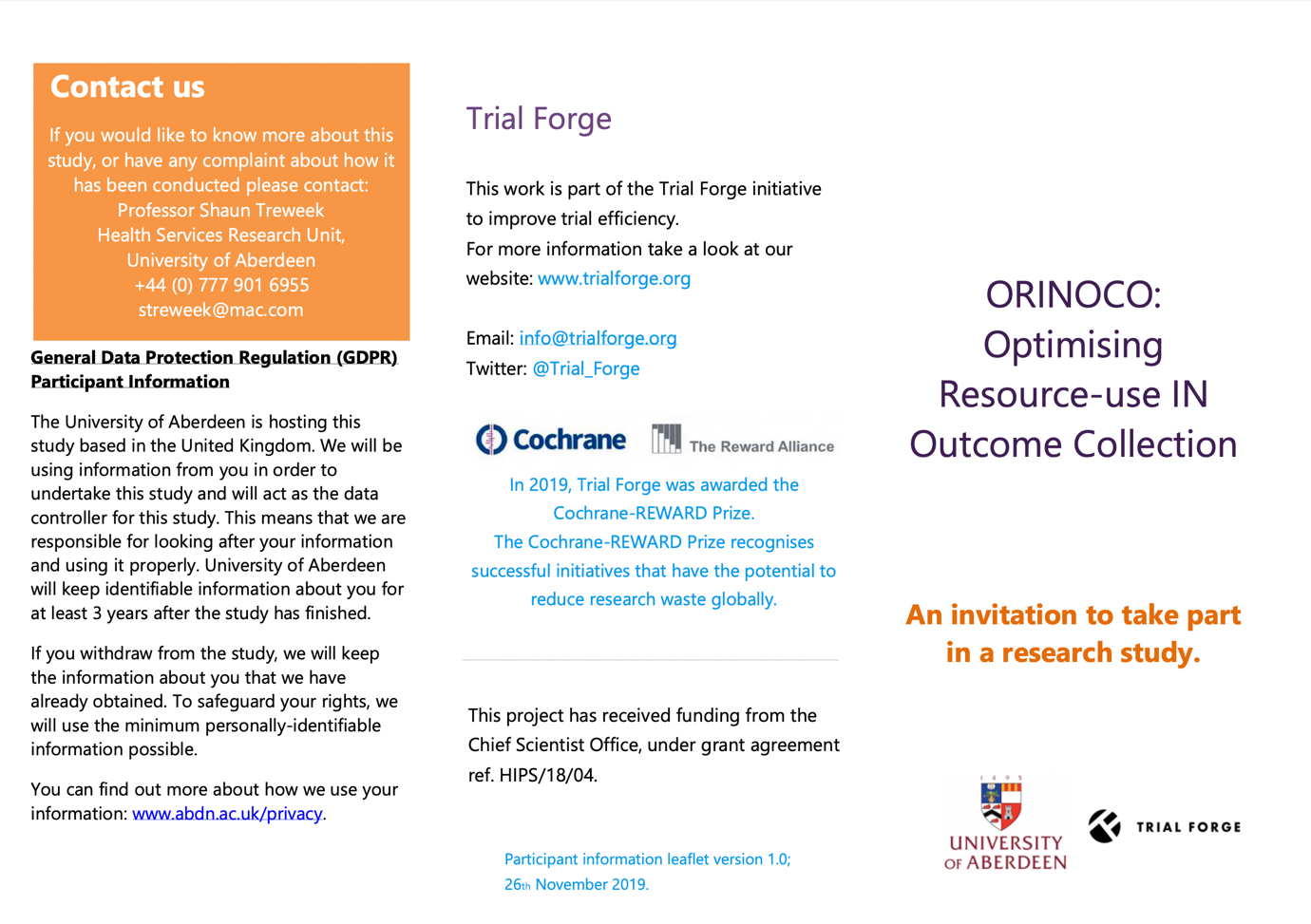


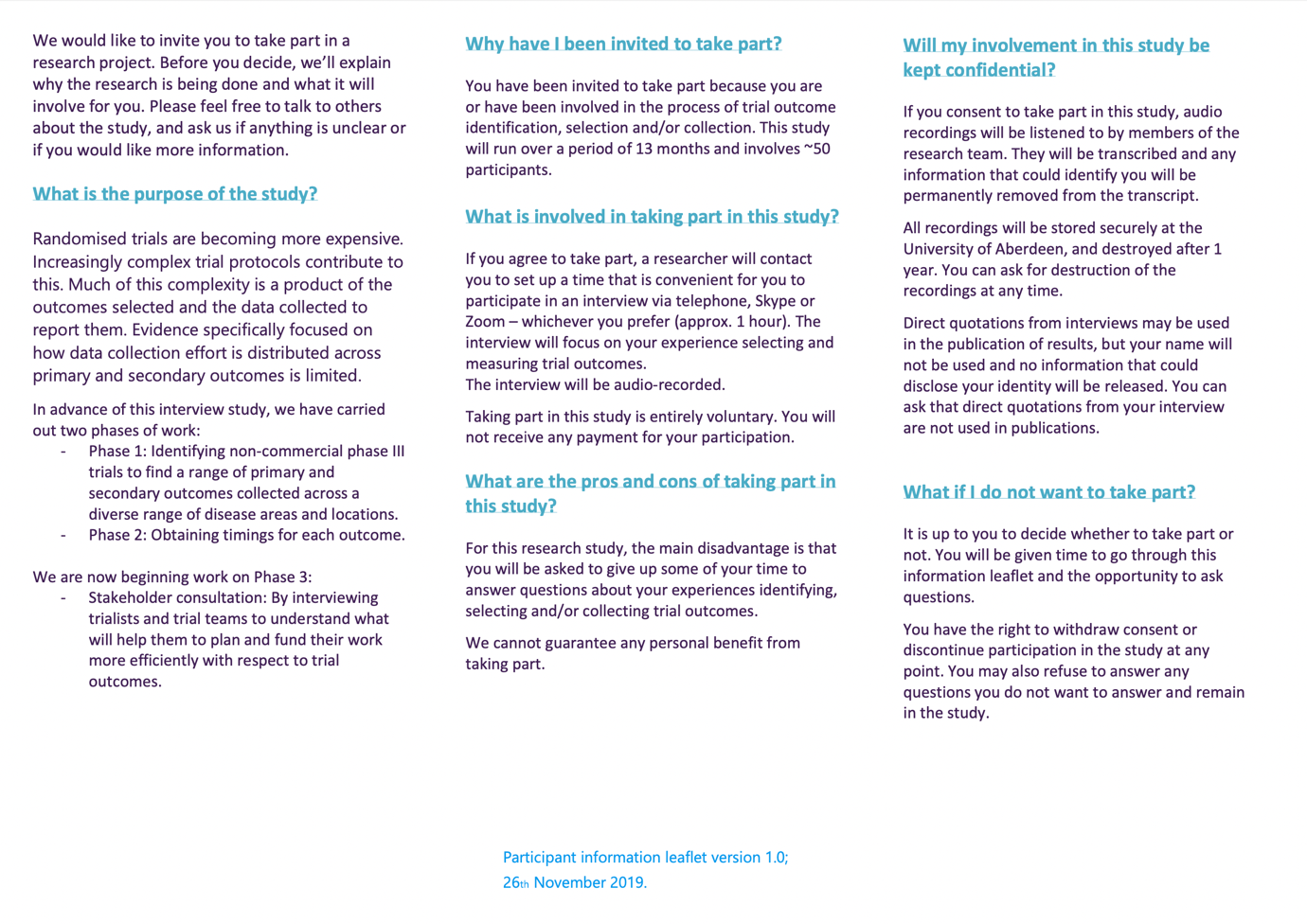

Supplement: Supplementary file 1 — Supplementary Material 1 [file 12874_2023_2054_MOESM1_ESM.docx]
