# Supplementary material for: Exploring the perspectives of selectors and collecters of trial outcome data: an international qualitative study
Source: BMC Med Res Methodol. 2023 Oct 11;23:229. doi: 10.1186/s12874-023-02054-9 (PMC10568821; doi:10.1186/s12874-023-02054-9)
Supplement: Supplementary file 2 — Supplementary Material 2 [file 12874_2023_2054_MOESM2_ESM.docx]

# ORINOCO Interview topic guide

Version 1.0, 26^th^ November 2019.

This topic guide will be used in a flexible manner to generate discussion between trial staff interviewees and the researcher. This topic guide may be subject to refinement.

My name is Heidi, and I am a researcher at the University of Aberdeen. Thank you for agreeing to participate in this interview. The general aim of this study is to help us understand more about outcome selection in trials.

I have a number of questions I’m going to ask you, and I’d like you to give them some thought and answer frankly. We are interested in your experiences and views so there are no right or wrong answers. All information collected will be strictly confidential. Our interview today will be recorded. The audio tapes will only be used for transcribing and analysing data.

How does that sound?

Do you have any questions for me before we start?

Do I have your consent to get started?

--------------------------------------------------------------------------------------------------------------------

- First, I would like to start off by asking you to tell me a little bit about yourself. So, can we start off with you telling me your job title, how long you have been in that role and what your role entails.
- To what extent do you see selecting outcomes for trials as part of your current professional role? (Prompt: what are those roles?)
- How confident are you in selecting outcomes for trials?
- Think of a recent example where you’ve worked on selecting a trial’s outcomes
  - Who else is involved in this process? How do the views of other colleagues affect your approach in selecting trial outcomes? (Prompt: what about clinicians, statisticians, trial managers, PPI partners, CTU directors, peer reviewers (grant application stage), ethics committees etc – are there any other people who might influence this process?)
  - How do you know you’ve chosen the ‘right’ outcome(s)? What is your interpretation of ‘successfully’ identifying outcomes for trials you’re involved with?
  - How easy/difficult is it to successfully identify outcomes for the trials you design?
  - What problems/difficulties do you encounter? When selecting outcomes for trials?
  - What would help you overcome these problems/difficulties?
  - Has past experience influenced how you select trial outcomes? (Prompt: how?)
  - Do you use any protocols, policies, or guidelines to guide your discussions for selecting trial outcomes? (Prompt: if so, which ones?)
  - If yes, do you think these are useful?
  - If no, is there any reason that you do not use guidelines? (Prompt for views on guidelines: do you think they’re a waste of time? Why?)
  - Are there strategies you currently use to assist trial outcome selection? (Prompt: formal or informal strategies, or personal methods? Can you think of any recent examples?)
  - (Following recent example) What would you do differently, what worked well? Are there any other strategies or ways of working that you might try next time?
- Now let’s think a bit more about the detail of how those outcomes that have been selected are collected.
  - Can you tell me a bit about the process of deciding on how outcomes are measured? (method of collection, time points, location etc – not what is measured, how) (Prompts: validated outcome measures versus trial-specific outcome measurement tools, PROMs)
  - When you’re deciding how outcomes are going to be measured, what factors do you take into account? (Prompts: budget, resource availability, potential for missing data, data quality, data volume)
- How would you feel about a resource that could tell you how long specific outcomes might take to collect?
  - If positive, what other information would you want from something like that?
  - If negative, tell me a bit about why you don’t like the sound of or wouldn’t use a resource like this?
- Let’s finish up by fast-forwarding to the end of the trial – you’ve collected your primary and secondary outcome data, what next?
  - Are you able to estimate what % of primary and secondary outcome data are published in your experience?
  - When you’re publishing the trial how do you decide which data to publish?
  - Can you tell me a bit about where the unpublished data are used?

They all the questions I have for you, is there anything else you’d like to talk about or expand on?
Thank you very much for your time.
